# Supplementary material for: The adoptive transfer of BCG-induced T lymphocytes contributes to hippocampal cell proliferation and tempers anxiety-like behavior in immune deficient mice
Source: PLoS One. 2020 Apr 2;15(4):e0225874. doi: 10.1371/journal.pone.0225874 (PMC7117742; doi:10.1371/journal.pone.0225874)
Supplement: S1 Data — (DOCX) [file pone.0225874.s001.docx]

**Highlights**

BCG vaccination affects splenic effector/memory T lymphocyte subsets in BALB/c wild-type mice.

The BCG-induced T lymphocytes contribute to hippocampal neurogenesis and behavior in immune deficient mice.

This work supports the opinion that adaptive immune processes could regulate brain functioning by T lymphocytes.
